# Gender differences in clinical characteristics of patients with non‐cystic fibrosis bronchiectasis in different age groups in northern China

**DOI:** 10.1111/crj.13596

**Published:** 2023-02-11

**Authors:** Yu‐yan Zhou, Yu‐hong Wang, Si‐qi He, Wan‐ying Wang, Xiao‐yue Wang, De‐shuai Li, Xiao‐ting Chen, Xiao‐kai Feng, Xiao‐ning Bu

**Affiliations:** ^1^ Department of Respiratory and Critical Care Medicine, Beijing Institute of Respiratory Medicine and Beijing Chaoyang Hospital Capital Medical University Beijing China; ^2^ Department of Respiratory and Critical Care Medicine, Beijing Tongren Hospital Capital Medical University Beijing China; ^3^ Department of Respiratory and Critical Care Medicine, Beijing Tiantan Hospital Capital Medical University Beijing China

**Keywords:** bronchiectasis, gender differences, non‐cystic fibrosis, sex

## Abstract

**Introduction:**

Patient gender has clinical and prognostic implications in non‐cystic fibrosis bronchiectasis, yet the potential effect of gender on clinical characteristics of patients with non‐cystic fibrosis bronchiectasis is still unclear.

**Objectives:**

This study aimed to investigate the gender differences in clinical characteristics of patients with bronchiectasis in different age groups in northern China.

**Methods:**

A total of 777 patients diagnosed with bronchiectasis were retrospectively included in Beijing Chaoyang Hospital and divided into two groups by gender: the male group and the female group. Each group was then subdivided into two according to their age (≤65 and >65 years). Gender differences in clinical characteristics were compared in all patients with bronchiectasis in the two age groups, respectively.

**Results:**

A total of 777 bronchiectasis patients were included. Of these patients, the prevalence of female non‐smokers was substantially higher than that of male non‐smokers (94.0% vs. 36.8%). There were gender differences in etiology of bronchiectasis, with more post‐measles and connective tissue disease in females (*p* = 0.006 and 0.002 separately) and more chronic obstructive pulmonary disease (COPD) in males (*p* < 0.001). The male group had a significantly higher C‐reactive protein (CRP) on admission (*p* = 0.03). Female patients showed a higher forced expiratory volume in 1 s as percentage of predicted volume (FEV1%pred) and forced vital capacity rate of 1 s (FEV1/FVC) (*p* < 0.001), lower partial pressure of carbon dioxide (PaCO_2_) (*p* = 0.04) and hospital costs (*p* = 0.02) than males, and a higher prevalence of infection with *Pseudomonas aeruginosa* in >65‐year‐old group (*p* = 0.019).

**Conclusions:**

There were many differences between male and female patients in smoking status, etiology, lung function, blood gas analysis, and hospital costs in all patients or different age groups.

## Graphical abstract

The association between gender and clinical characteristics of non‐cystic fibrosis bronchiectasis was investigated in attempt to improve better management. Our findings demonstrate that significant disparities are found between the male group and the female group in terms of the clinical characteristics of bronchiectasis.

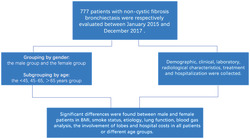

**Abbreviations**
BMI: body mass index
COPD: chronic obstructive pulmonary disease
CRP: C‐reactive protein
FEV_1_%pred: forced expiratory volume in 1 s as percentage of predicted volume
FEV_1_/FVC: forced vital capacity rate of 1 s
PaCO_2_
: partial pressure of carbon dioxide

## 1 INTRODUCTION

Non‐cystic fibrosis bronchiectasis (NCFB) (henceforth referred to as bronchiectasis) is a chronic respiratory disease characterized by abnormal and permanent dilatation of the bronchi associated with chronic cough, sputum production, and recurrent respiratory infection.^1^, ^2^, ^3^ A cross‐sectional survey reported by Zhou et al. showed that the prevalence of bronchiectasis in residents aged 40 years and above in 7 provinces of China is 1.2% (135/10 811)^4^; however, these underestimated data are still higher than those of some European countries (67/100 000 in Germany).^5^

Increasing epidemiological and biological data suggest the role of gender in disease pathogenesis and outcomes.^6^ The impact of gender on airway disease has piqued researchers' curiosity. Gender differences in disease prevalence, severity, progression, and outcome have been well described in individuals with chronic obstructive pulmonary disease (COPD)^7^ and cystic fibrosis (CF).^8^ Females with COPD are more likely to have a chronic bronchitis phenotype, suffer from less cardiovascular comorbidity, have more concomitant depression and osteoporosis, and have a better outcome with acute exacerbations.^7^ Further, although a higher prevalence among males is observed in CF, females who have CF are reported to show increased severity, worse clinical outcomes, poorer lung function, and a survival disadvantage than males.^8^ Gender differences in NCFB are also clinically evident and should be taken into account. Previous research has found gender‐related differences in the clinical features of bronchiectasis^9^; however, most of these studies have been restricted to the small sample size and, to date, there are still few studies that have investigated the association between gender and clinical features of patients with NCFB in different age groups.

As a result, the aim of this study was to investigate gender differences in clinical, laboratory, radiological characteristics, treatment, length of hospital stay, and hospital costs of patients with bronchiectasis in different age groups. We anticipate that our research can contribute to a better understanding of the association between gender and bronchiectasis, allowing clinicians to make better decisions and develop gender‐specific treatment strategies.

## 2 METHODS

### 2.1 Study design

The clinical data of all bronchiectasis patients hospitalized for exacerbation at Beijing Chaoyang Hospital between January 2015 and December 2017 were retrospectively collected and analyzed. The definition of NCFB was according to clinical practice guidelines of the British Thoracic Society (BTS)^10^ in accordance with a high‐resolution computed tomography (HRCT) and clinical signs. Included patients were divided into male patients and female patients based on gender, and each group was subdivided into two groups by age (≤65 and >65 years). The gender differences in clinical characteristics were respectively compared in all patients and in the two age groups. The Ethics Committee of Beijing Chaoyang Hospital (2017‐KE‐92) approved the collection of clinical data from the patients. The studies were conducted in accordance with the relevant guidelines and regulations. Written informed consent for publication was obtained from all participants.

### 2.2 Variables

The clinical data were collected from the original medical records at Beijing Chaoyang Hospital. The variables included anthropometry (gender, age, body mass index [BMI], and smoking history), clinical symptoms on admission (cough, sputum volume, hemoptysis, and Medical Research Council [MRC] dyspnea score), etiology of bronchiectasis (idiopathic, post‐measles, post‐whooping cough, post‐pulmonary tuberculosis, COPD, asthma, connective tissue disease, and allergic bronchopulmonary aspergillosis [ABPA]), inflammation marker (C‐reactive protein [CRP] on admission), lung function indices (forced expiratory volume in 1 s as percentage of predicted volume [FEV_1_%pred] and forced vital capacity rate of 1 s [FEV_1_/FVC]), arterial blood gas (pH, partial pressure of oxygen [PaO_2_], and partial pressure of carbon dioxide [PaCO_2_]), infection with organisms (*Pseudomonas aeruginosa* or other organisms), radiographic manifestations (affected lobes and location), treatment (antibiotics, expectorant, cough medicine, oxygen, and non‐invasive ventilation), length of stay, and hospital costs from admission to discharge. All the assessments were performed in accordance with relevant testing standards.

### 2.3 Statistics

Statistical analysis was performed with SPSS 25.0. Descriptive statistics of the median with 95% confidence intervals were used for quantitative variables, absolute frequencies, and percentages for qualitative data. Normality was tested by the Kolmogorov–Smirnov test and the Shapiro–Wilk test. Subgroup comparisons were performed by the Student *t*‐test for normally distributed variables, the Mann–Whitney *U* test for non‐normally distributed variables, and the chi‐square test for qualitative variables. Multivariate linear regression models were used for evaluating the relationship between lung function and gender, smoking status, and COPD to exclude potential confounders. A comparison‐wise significance level of 5% was used.

## 3 RESULTS

A total of 777 patients with NCFB were included in this study, with 378 (48.6%) males and 399 (51.4%) females. The two age groups (≤65 and >65 years) included 423 and 354 patients, respectively. Demographic characteristics, clinical symptoms, and disease duration for males and females are summarized in Table 1. Significant differences in smoking status were observed between males and females in all patients (*p* < 0.001). The prevalence of female non‐smokers was substantially higher than that of male non‐smokers (94.0% vs. 36.8%), and the results of the two age groups were consistent with the overall situation. Age, BMI, cough, sputum volume, hemoptysis, MRC dyspnea score, and disease duration were comparable between the male group and the female group.

**TABLE 1:** Demographic characteristics, clinical symptoms and disease duration according to gender and age.

Variables	Total (*N* = 777)		*p*	≤65 years (*n* = 423)		*p*	>65 years (*n* = 354)		*p*
	Male	Female		Male	Female		Male	Female	
Demographics									
Subject	378 (48.6)	399 (51.4)		196 (46.3)	227 (53.7)		182 (51.4)	172 (48.6)	
Age (years)	64.8 (55.5–72.0)	63.5 (56.0–70.3)	0.27	55.9 (49.8–61.5)	57.4 (50.7–61.8)	0.206	72.5 (68.8–77.4)	71.4 (67.9–77.0)	0.19
BMI (kg/m^2^)	23.5 (20.1–26.3)	22.8 (20.7–25.4)	0.10	23.4 (20.1–26.0)	22.3 (20.5–24.7)	0.077	23.9 (20.1–27.0)	23.1 (20.9–26.0)	0.70
Smoking status			<0.001			<0.001			<0.001
Never	139 (36.8)	375 (94)		84 (42.9)	221 (97.4)		55 (30.2)	154 (89.5)	
Current	158 (41.8)	14 (3.5)		42 (21.4)	3 (1.3)		88 (48.4)	11 (6.4)	
Past	81 (21.4)	10 (2.5)		70 (35.7)	3 (1.3)		39 (21.4)	7 (4.1)	
Clinical symptom									
Cough	367 (97.1)	380 (95.2)	0.18	187 (95.4)	211 (93.0)	0.285	180 (98.9)	169 (98.3)	0.68
Sputum volume			0.10			0.398			0.12
<10 mL	246 (65.1)	236 (59.1)		121 (61.7)	130 (57.3)		125 (68.7)	106 (61.6)	
10–150 mL	109 (28.8)	135 (33.8)		59 (30.1)	82 (36.1)		50 (27.5)	53 (30.8)	
>150 mL	23 (6.1)	28 (7.0)		16 (8.2)	15 (6.6)		7 (3.8)	13 (7.6)	
Hemoptysis	135 (35.7)	130 (32.6)	0.36	69 (35.2)	79 (34.8)	0.931	66 (36.3)	51 (29.7)	0.19
MRC dyspnea score	1 (0–2)	1 (0–2)	0.51	1 (0–2)	1 (0–2)	0.845	2 (1–2)	2 (0–2)	0.83
Disease duration (days)	8.0 (1.0–21.0)	9.5 (1.0–30.0)	0.30	6.5 (1.0–20.0)	6.0 (1.0–20.0)	0.599	10.0 (1.0–30.0)	10.0 (2.0–30.0)	0.28

*Note*: Data are presented as *n* (%) or median (95% confidence intervals).

Abbreviations: BMI, body mass index; MRC, Medical Research Council.

Idiopathic (38.4%), asthma (16.9%), and post‐pulmonary tuberculosis (13.0%) were the most common pulmonary etiologies among the 777 patients with NCFB. It can be seen from the data in Table 2 that there were significant differences in measles, COPD, and connective tissue disease of total patients with NCFB between the male group and the female group. Female patients with NCFB caused by measles had a substantially higher rate than male patients (*p* = 0.006) and in the age group of ≤65 years (*p* = 0.012). The male group had a substantially greater prevalence of COPD‐related bronchiectasis than the female group, both in the age groups of ≤65 and >65 years (*p* = 0.001 and *p* < 0.001, respectively). For connective tissue disease, female patients were more than males in all patients (*p* = 0.002) and in the age groups of ≤65 years (*p* = 0.037) and >65 years (*p* = 0.02). In addition to the above mentioned factors, female patients with idiopathic bronchiectasis were more than males in the age groups of ≤65 and >65 years (*p* = 0.028 and *p* < 0.001, respectively). The percentage of patients with NCFB caused by post‐pulmonary tuberculosis was higher in the female group of patients less than 65 years compared with the male group (*p* = 0.041). However, the prevalence of male patients with NCFB caused by post‐pulmonary tuberculosis was higher than that of female patients over 65 years old (*p* = 0.01). There were no significant differences between males and females in terms of the prevalence of other etiologies of bronchiectasis.

**TABLE 2:** The etiology of NCFB according to gender and age.

Variables	Total (*N* = 777)		*p*	≤65 years (*n* = 423)		*p*	>65 years (*n* = 354)		*p*
	Male	Female		Male	Female		Male	Female	
Etiology									
Idiopathic	139 (36.8)	159 (39.8)	0.38	87 (44.4)	77 (33.9)	0.028	52 (28.6)	82 (47.7)	<0.001
Post‐measles	3 (0.8)	15 (3.8)	0.006	1 (0.5)	10 (4.4)	0.012	2 (1.1)	5 (2.9)	0.27
Post‐whooping cough	1 (0.3)	2 (0.5)	0.60	1 (0.5)	2 (0.9)	0.650	0 (0)	0 (0)	NS
Post‐pulmonary tuberculosis	52 (13.8)	49 (12.3)	0.54	14 (7.1)	30 (13.2)	0.041	38 (20.9)	19 (11)	0.01
COPD	49 (13)	9 (2.3)	<0.001	14 (7.1)	2 (0.9)	0.001	35 (19.2)	7 (4.1)	<0.001
Asthma	65 (17.2)	66 (16.5)	0.81	41 (20.9)	38 (16.7)	0.272	24 (13.2)	28 (16.3)	0.41
Connective tissue disease	3 (0.8)	17 (4.3)	0.002	3 (1.5)	12 (5.3)	0.037	0 (0)	5 (2.9)	0.02
ABPA	3 (0.8)	5 (1.3)	0.73	1 (0.5)	4 (1.8)	0.235	2 (1.1)	1 (0.6)	0.60
Other	63 (16.7)	77 (19.3)	0.34	34 (17.3)	52 (22.9)	0.157	29 (15.9)	25 (14.5)	0.71

*Note*: Data are presented as *n* (%) or median (95% confidence intervals).

Abbreviations: ABPA, allergic bronchopulmonary aspergillosis; COPD, chronic obstructive pulmonary disease; NCFB, non‐cystic fibrosis bronchiectasis; NS, not significant.

A comparison of the laboratory, radiological characteristics between males and females is summarized in Table 3. Of all patients, males had significantly higher CRP, worse lung function, lower pH, higher PaCO_2_, and more hospital costs. Moreover, there were no significant differences in the PaO_2_, the involved lobes of bronchiectasis, treatment, and the length of hospitalization between the female group and the male group. The CRP of male patients was significantly higher than that of females in all patients (*p* = 0.03). Lung function parameters were available in 762 patients (372 males and 390 females). The FEV_1_%pred and FEV_1_/FVC of females with NCFB were significantly higher than those of males with NCFB in all patients, in the age groups of ≤65 and >65 years. It was known that smoking and COPD were associated with worse lung function. Further, there were significant differences in smoking status and COPD between the male group and the female group in this study. Thus, we considered that smoking and COPD were potential confounders for the divergence of lung function between the male group and the female group. Male patients with NCFB are more likely to smoke than female patients, which may account for the gender differences in lung function parameters. In the multivariate linear regression models, there was a linear relationship between gender, COPD, and lung function (*p* < 0.001). It means that there was still a significant gender difference in the lung function of patients with bronchiectasis after excluding confounders (smoking and COPD).

**TABLE 3:** Laboratory, functional status, radiological characteristics according to gender and age.

Variables	Total (*N* = 777)		*p*	≤65 years (*n* = 423)		*p*	>65 years (*n* = 354)		*p*
	Male	Female		Male	Female		Male	Female	
CRP (mg/dL)	0.93 (0.33–2.99)	0.66 (0.29–2.14)	0.03	0.5 (0.2–2.6)	0.5 (0.2–1.3)	0.21	1.04 (0.42–2.83)	0.70 (0.30–2.50)	0.20
Functional status									
FEV1%pred	51.9 (35.0–81.2)	70.2 (47.5–87.8)	<0.001	51.1 (33.1–83.3)	70.8 (46.3–86.3)	<0.001	54.6 (36.3–77.8)	69.5 (49.0–90.9)	<0.001
FEV1/FVC	58.7 (45.8–71.4)	66.3 (54.2–74.8)	<0.001	59.4 (45.4–72.2)	67.4 (54.7–75.3)	<0.001	58.7 (45.9–70.3)	65.0 (54.2–74.1)	<0.001
Blood gas analysis									
pH	7.40 (7.38–7.43)	7.41 (7.39–7.43)	0.001	7.4 (7.3–7.4)	7.4 (7.3–7.4)	0.01	7.40 (7.38–7.43)	7.41 (7.39–7.43)	0.10
PaO_2_ (mmHg)	76.1 (68.0–84.0)	77.3 (68.0–85.9)	0.25	76.0 (67.7–83.5)	78.0 (69.0–85.8)	0.18	77.0 (69.0–85.0)	77.1 (67.0–87.0)	0.75
PaCO_2_ (mmHg)	42.0 (39.0–46.0)	41.2 (38.2–45.0)	0.04	43.0 (39.0–47.0)	42.0 (39.0–45.0)	0.04	42.0 (38.0–46.0)	41.0 (37.6–45.0)	0.31
Involvement of lobes									
Right upper lobe	116 (30.7)	124 (31.1)	0.91	66 (33.6)	80 (35.2)	0.73	50 (27.5)	44 (25.6)	0.69
Right middle lobe	146 (38.6)	170 (42.6)	0.26	83 (42.3)	102 (44.9)	0.59	63 (34.6)	68 (39.5)	0.34
Right lower lobe	191 (50.5)	185 (46.4)	0.25	102 (52.0)	111 (48.9)	0.51	89 (48.9)	74 (43.0)	0.27
Left upper lobe	62 (16.4)	83 (20.8)	0.12	34 (17.3)	46 (20.2)	0.44	28 (15.4)	37 (21.5)	0.14
Lingual	109 (28.8)	120 (30.1)	0.71	64 (32.6)	72 (31.7)	0.83	45 (24.7)	48 (27.9)	0.50
Left lower lobe	219 (57.9)	215 (53.9)	0.26	113 (57.6)	121 (53.3)	0.37	106 (58.2)	94 (54.7)	0.54

*Note*: Data are presented as *n* (%) or median (95% confidence intervals).

Abbreviations: CRP, C‐reactive protein; FEV1%pred, forced expiratory volume in 1 s as percentage of predicted volume; FEV1/FVC, forced vital capacity rate of 1 s; PaCO_2_, partial pressure of carbon dioxide; PaO_2_, partial pressure of oxygen.

Results of blood gas analysis were available in 715 patients (349 males and 366 females). Though male patients had lower pH and higher PaCO_2_ in total and in the age group of ≤65 years, there were no significant differences in pH and PaCO_2_ in the age group of >65 years. The involved lobes of bronchiectasis according to the HRCT were also evaluated in our study. There was no significant difference in the involved lobes between the female group and the male group among all the patients and between the two age groups.

**FIGURE 1:**
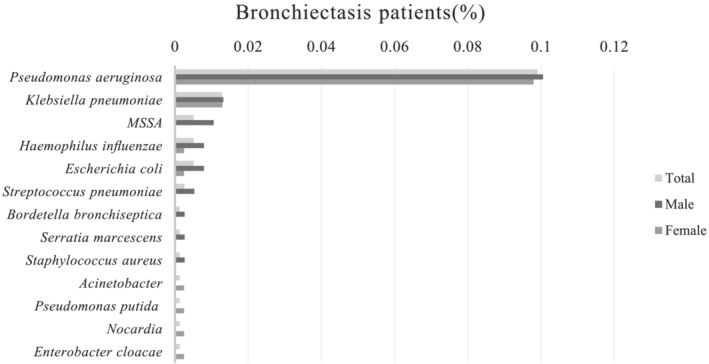
Potential pathogenic microorganisms present in sputum samples of bronchiectasis patients. MSSA, methicillin‐sensitive *Staphylococcus aureus* .

Results of sputum cultures were available in all patients, and there were 108 patients (58 males and 50 females) with sputum positive. Figure 1 shows the potential pathogenic microorganisms present in sputum samples of bronchiectasis patients. The most common pathogens for males were *P. aeruginosa* (10.1%), *Klebsiella pneumoniae* (1.3%), and methicillin‐sensitive *Staphylococcus aureus* (MSSA) (1.1%), and those for females were *P. aeruginosa* (9.8%) and *K. pneumoniae* (1.3%). In the age group of >65 years, the proportion of females who were infected with *P. aeruginosa* was higher than that of males (89.47% vs. 54.55%, *p* = 0.019). There were no gender differences in the sputum culture of bronchiectasis patients among all patients and the two age groups separately.

A comparison of the treatment and the hospitalization between males and females is summarized in Table 4. As for treatment, the data indicated that there was no significant difference in use of antibiotics, expectorant, oxygen, and non‐invasive ventilation between the male group and the female group. However, the proportion of males who use cough medicine was higher than that of females both in total patients (*p* = 0.05) and in the age group of ≤65 years (*p* = 0.04). In terms of hospitalization, no significant difference was found in the length of hospitalization; however, the hospital costs were higher in males than females in total (*p* = 0.02) and in the age group of ≤65 years (*p* = 0.01).

**TABLE 4:** Treatment and hospitalization according to gender and age.

Variables	Total (*N* = 777)		*p*	≤65 years (*n* = 423)		*p*	>65 years (*n* = 354)		*p*
	Male	Female		Male	Female		Male	Female	
Treatment									
Antibiotics	325 (86.0)	329 (82.5)	0.18	164 (83.6)	189 (83.2)	0.90	161 (88.5)	140 (81.4)	0.06
Expectorant	180 (47.6)	194 (48.6)	0.78	98 (50.0)	117 (51.5)	0.75	82 (45.1)	77 (44.8)	0.96
Cough medicine	96 (25.4)	78 (19.5)	0.05	57 (29.0)	47 (20.0)	0.04	39 (21.4)	31 (18.0)	0.42
Oxygen	251 (66.4)	249 (62.4)	0.25	126 (64.2)	139 (61.2)	0.51	125 (68.7)	110 (64.0)	0.35
Non‐invasive ventilation	7 (1.9)	4 (0.1)	0.32	5 (2.5)	3 (1.3)	0.35	2 (1.1)	1 (0.6)	>0.99
Hospitalization									
Length of hospitalization (days)	10 (7–13)	9 (7–12)	0.41	9.0 (7.0–13.0)	9.0 (7.0–12.0)	0.23	10 (8–13)	10 (8–13)	0.75
Hospital costs (RMB)	11 355 (9072–16 174)	10 754 (8540–14 183)	0.02	11 380.4 (8779.7–15 034.6)	10 252.0 (8049.0–13 223.0)	0.01	11 318 (9219–16 850)	11 728 (9467–15 314)	0.71

## 4 DISCUSSION

The aim of this study was to investigate the gender differences in clinical characteristics of patients with NCFB in different age groups in northern China. Our study demonstrated that there were many significant differences such as smoking history, etiology, initial CRP level, lung function, blood gas analysis, and hospital costs between male and female patients.

Smoking is a known cause of COPD.^11^ In our study, male patients with bronchiectasis were more likely to smoke than females. Consistent with previous research in Europe,^12^ COPD‐related bronchiectasis patients were seen more frequently among males than females over 45 years of age. As a result, our findings that more males over 65 years of age tended to have COPD‐related bronchiectasis than females probably are in line with the evidence that the proportion of male smokers was much higher than that of female smokers. However, previous studies demonstrate that women are more susceptible to cigarette smoking than males. Additionally, women smokers tended to experience a faster decline in lung function than males.^13^ Whether females are more likely to have COPD‐related bronchiectasis when excluding the potential confounders for smoking than males is still unknown.

Other common causes of NCFB include measles, connective tissue disease, and post‐pulmonary tuberculosis. Our findings demonstrated that there were also gender disparities in the etiology of NCFB such as measles, connective tissue disease, and post‐pulmonary tuberculosis in different age groups. In our study, the prevalence of NCFB caused by connective tissue disease was much higher in females than males. Additionally, connective tissue disease that may cause bronchiectasis was more often seen in women. However, little is known about whether a higher prevalence of connective tissue disease in women translates to a higher prevalence of bronchiectasis due to connective tissue disease in women. To date, there is a paucity of studies regarding the causality for the divergence in NCFB between males and females. Future research should expand the sample size and collect follow‐up data to accurately reflect the gender variations in etiology.

A variety of respiratory disorders differ by gender in the outcomes. Compared with males, females with CF tended to have poorer lung function, greater disease severity, and a lower survival rate across all age groups.^14^ It has been reported that males tended to have poorer lung function in bronchiectasis than females,^9^ which is the opposite in CF.^15^, ^16^ Our study demonstrated that males with bronchiectasis had poorer lung function across all age groups than females, which is consistent with the previous research. Yet gender differences in lung function among young patients with NCFB have not been obvious and remain to be investigated. A cohort study by Huang et al. showed that the COPD‐related bronchiectasis had a poorer lung function and severer airflow obstruction than bronchiectasis caused by other etiologies.^17^ Considering that poorer lung function observed in males may be associated with a higher prevalence of males in COPD‐related bronchiectasis, we used multivariate linear regression models to exclude the effect of COPD. Our study demonstrated that there was still a gender difference in lung function of patients with bronchiectasis after excluding confounders (smoking and COPD).

Martínez‐García et al. demonstrated that chronic colonization by *P. aeruginosa*, severe exacerbations, and systemic inflammation are the independent factors associated with lung function decline in patients with stable bronchiectasis.^18^ The level of CRP, as one of the inflammatory markers, was higher on admission of male patients with bronchiectasis than that of females in all patients. Females were more likely to be infected with *P. aeruginosa* than males in >65 years age group. Considering that males may be more likely to be infected with other bacteria, future research should be performed to explore the contributing factors and mechanisms of gender differences in lung function of bronchiectasis patients such as types and distribution of colonized organisms.

As for blood gas analysis, male patients with bronchiectasis had lower pH and higher PaCO_2_ of total patients. These results implied that male patients with bronchiectasis have more severe CO_2_ retention. It was consistent with the observation that male patients with bronchiectasis have poorer lung function than females.

Our study showed that there was no significant difference in the involved lobes between the female group and the male group among all the patients and between the two age groups. Izhakian et al. demonstrated that, in NCFB, different pathogens were associated with different lobar distributions and that *P. aeruginosa* was the dominant species isolated in the right upper lobe.^19^ The difference between our study and previously published studies may be due to the lack of the younger group. Because there were no significant differences in infection of pathogens in young patients, future research should further consider the potential mechanism of gender differences in lobe involvement.

Our study demonstrated that the hospital costs of male patients with bronchiectasis are higher than those of females in total patients and less than 65 years of age. As our study was a retrospective investigation, it was difficult to assess the disease severity of bronchiectasis. However, lung function is a crucial factor in assessing the severity of bronchiectasis.^3^ Poorer lung function might imply worse severity of bronchiectasis, which correlates with the higher cost of hospitalization. Furthermore, lengths of hospital stay and hospital costs will be confounded by the decision bias of the physician responsible for each patient's care and will be significantly influenced by the reason for that admission. Future research should further eliminate the bias caused by these confounding factors.

Pulmonary infection has been shown as a substantial risk factor for the development of bronchiectasis.^3^ *P. aeruginosa* is recognized as a marker of bronchiectasis severity and is correlated with increased mortality, hospital admissions, and exacerbations.^20^, ^21^ In our study, *P. aeruginosa* was the most common pathogen for males and females, and *K. pneumoniae* was the second common pathogen. The proportion of female patients who were infected with *P. aeruginosa* was higher than that of males in elderly patients. There was no significant difference in bacterial colonization between the female group and the male group in all patients and other age groups. Additionally, the prevalence of male patients infected with *P. aeruginosa* was comparable with that of female patients in all patients. Previous research has reported that *P. aeruginosa* is the primary bacterium that infects patients with bronchiectasis.^22^, ^23^ In a previous retrospective study, *P. aeruginosa* was found to be the primary pathogen in females with bronchiectasis, whereas males were dominated by *Haemophilus influenzae*.^24^ However, the lack of specialized research on the gender differences in bronchiectasis airway microbiology and microbiome composition is apparent.^6^ Determining the gender differences in bronchiectasis airway microbiome composition could provide a theoretical basis for clinically individualized therapy that can help patients to improve clinical symptoms, reduce exacerbation frequency, and lower the microbial airway load.

There are several limitations to the current study, which should be mentioned. First, a retrospective study from a single center could inevitably result in a selection bias. Further, data are incomplete for follow‐up. Thus, we did not explore the gender difference of patients in the improvements of prognosis. Finally, the period of bronchiectasis is divided into stable and acute exacerbations. The clinical data in the current study were collected from patients with bronchiectasis during the period of acute exacerbation. In the future, data from stable bronchiectasis can be further collected for gender difference research, which could provide a theoretical basis for therapy of stable bronchiectasis.

In conclusion, we found that male patients with bronchiectasis had poorer lung function, higher CRP level, and higher hospital costs than female patients. The proportion of female patients who were infected with *P. aeruginosa* was higher than that of males in elderly patients. No significant differences were observed in clinical symptoms, treatment, and the involvement of lobes. Further research exploring the mechanisms behind this disparity is necessary and may illustrate potential therapeutic options to improve prognosis.

## AUTHOR CONTRIBUTIONS

Z. Y. had full access to all the data in the study and takes responsibility for the integrity of the data and the accuracy of the data analysis. Z. Y. and B. X. contributed to the conception and design of the study; W. Y., W. W., H. S., W. X., L. D., C. X., and F. X. contributed to the performing of the study; Z. Y. and B. X. contributed to the data analysis and interpretation; Z. Y. and B. X. contributed to the writing of the paper; and Z. Y., W. Y., H. S., B. X., W. W., W. X., L. D., C. X., and F. X. contributed to the writing, review, and approval of the manuscript and made substantial contributions to important intellectual content.

## CONFLICT OF INTEREST STATEMENT

The authors have declared that there is no conflict of interest related to the manuscript.

## ETHICS STATEMENT

Written informed consent for publication was obtained from all participants. The Ethics Committee of Beijing Chaoyang Hospital (2017‐KE‐92) approved the collection of clinical data from the patients. The data are with the authors and will be available upon reasonable request.

## Data Availability

The data that support the findings of this study are available from the corresponding author upon reasonable request.
